# Beyond the Diagnosis: Lived Experiences of Persons with Spinal Cord Injury in a Selected Town in Ghana

**DOI:** 10.1155/2019/9695740

**Published:** 2019-01-16

**Authors:** Abdul-Ganiyu Fuseini, Patience Aniteye, Afizu Alhassan

**Affiliations:** ^1^Department of Nursing, School of Allied Health Sciences, University for Development Studies, Tamale-Ghana. P.O. Box, TL 1883, Tamale, Ghana; ^2^Department of Community Health Nursing, School of Nursing, University of Ghana, P.O. Box LG. 43, Legon, Accra, Ghana; ^3^Nursing and Midwifery Training College, Kpembe, P.O. Box SL98, Salaga, Ghana

## Abstract

**Background:**

Although several studies have been conducted on the lived experiences of persons with spinal cord injury (SCI) in high income countries, there is no published data on such experiences in Ghana. The purpose of this study was to explore the lived experiences of persons with SCI in the Tamale Metropolis of the Northern Region of Ghana.

**Material and Methods:**

A qualitative descriptive design involving thirteen participants was conducted at the Tamale Metropolis-Ghana. A purposive sampling technique was used to recruit participants, using the Neurosurgical Unit of the Tamale Teaching Hospital as an outlet for recruitment of the sample. Data was gathered mainly through face-to-face in-depth interviews. The data was analyzed concurrently with data collection, using thematic content analysis. Ethical approval was obtained for the study from the Noguchi Memorial Institute for Medical Research and the research unit of the Tamale Teaching Hospital.

**Results:**

The three main themes that emerged from the data during analysis were “physical effects,” “psychological effects,” and “social issues.”* Conclusion*. The findings from the study suggest that SCI is a life threatening condition and that persons with SCI grapple with a myriad of physical symptoms that range from chronic pain and paralysis of lower and/or upper limbs, to bladder and bowel incontinence. These physical symptoms have significant psychological and social effects on the functioning of the affected persons.

## 1. Background

Spinal Cord Injury (SCI) is a devastating life threatening condition that affects every aspect of life, including the physical, social, and psychological health of affected persons [1–3]. The sudden onset of SCI is tragic and has a critical impact on the patient and significant others [3]. Despite the devastating nature of the condition, studies have reported a dramatic improvement in survival over the last 40 years, especially in high income countries [4, 5]. The improved survival rate and increased life expectancy amongst persons with SCI mean that spinal injured persons will live with a disability for longer periods of time, giving rise to new problems and responsibilities that can negatively impact their lives [6]. A number of studies have been conducted on the lived experiences of persons with SCI [7–10]. Most of these studies were conducted in North America, Europe, Asia, and other high income countries. Limited research has been conducted in low and middle income countries (LMIC) to explore the experiences of persons with SCI and the impact of these experiences on their functioning and general wellbeing. Also, there is evidence in the literature that persons with SCI have many negative experiences ranging from physical and psychological morbidities to community reintegration problems that negatively affect their functioning and general wellbeing [9, 11, 12]. Furthermore, the first author, based on his clinical experience in northern Ghana, observed that patients with SCI were faced with physical, psychological, and social problems. These problems have not been systematically explored to determine the effects of these experiences on the functioning and wellbeing of individuals with SCI. It is based on the above inferences that this study employed a qualitative approach to explore the lived experiences of persons with SCI in the Tamale Metropolis of Ghana. To the best of the authors' knowledge, there is no published data on the experiences of persons with SCI in Ghana.

## 2. Methods

### 2.1. Design

The study was completed as part of the requirements for a Master of Philosophy programme (Nursing) for the first author to explore the lived experiences of persons living with SCI. The study employed a descriptive qualitative design [13]. In order to ensure that, a sufficient number of participants that met the inclusion criteria for the study were recruited, and to achieve a broad understanding of the lived experiences of persons living with SCI, purposive sampling was used to recruit participants for the study.

### 2.2. Setting

The study was conducted in the Tamale Metropolis which is the largest city in the three northern regions of Ghana and the third largest metropolis in the country. The Metropolis is one of the 26 districts in the Northern Region. It is located in the central part of the Region and shares boundaries with the Sagnarigu District to the west and north, Mion District to the east, East Gonja to the south, and Central Gonja to the southwest. It has a population of 360,579 according to the 2010 Population and Housing Census. Unlike the southern part of Ghana where cars and minibuses called “trotro” are the major means of transport, the major means of transport for residents in the Tamale metropolis is with motor bicycles. This has led to high prevalence of cases of SCI according to anecdotal evidence at the Tamale Teaching Hospital.

### 2.3. Sampling and Procedure of Data Collection

Data for the study was collected mainly through face-to-face in-depth interviews. The purpose of the study was to explore the lived experiences of persons with SCI, and therefore interviews were the best data collection method to gain this understanding. The participants were recruited through the purposive sampling technique. Fliers specifying the purpose of the study were given to participants by nurses in the neurosurgical unit of the hospital. Participants who best met the inclusion criteria of having a SCI, being eighteen years of age or over, and ability to communicate in English or Dagbani (a local dialect) were contacted by the first author to explain the purpose of the study to them. The participants were subsequently recruited for the study following an informed consent. Interviews were conducted with the aid of an interview guide. The interview guide explored four major areas (background information; physical experiences; psychological experiences; and social experiences), through the use of twelve (12) open-ended questions. The questions were developed based on the objectives of the study and the research questions. The interviews were conducted by the first author in English and/or Dagbani (local dialect) because these were languages that the participants understood and could speak fluently. The interviews lasted for approximately 45 to 65 minutes and were tape-recorded with permission from the participants. The interviews were also conducted at the participants' preferred time and were completed in a private room to ensure privacy. The first author used flexible open-ended questions and intentional silence during the interviews to allow participants to express their thoughts. The first author also took field notes of all nonverbal communications during the data collection process to ensure that every aspect of the data was captured and to help in the analysis.

### 2.4. Data Management and Analysis

Data from the study was analyzed concurrently with data collection using content analysis [14]. At the end of each interview, the first author manually transcribed verbatim the audio-taped recording of the interview. The accuracy of the manual transcripts was checked by a colleague researcher who read the transcript and at the same time listened to the audio-taped recordings. For interviews that were conducted in the local dialect (Dagbani), the transcripts were translated into English by another person who speaks and writes Dagbani to ensure the accuracy of the translation. After all audio-recordings had been manually transcribed, the transcripts were printed and the data was then analyzed using thematic content analysis. Each printed transcript was read and reread many times to gain a sense of the whole and to become familiar with the content of the transcript. During the analysis, the researchers searched the transcript for similar ideas, thoughts, and words and these made up the thematic codes. Identified thematic codes were written on the hard copies of the transcripts. Each transcript was handled in this same manner, and new codes that emerged during the process were added until all the transcripts were coded. Following the coding, all information belonging to a code were copied and pasted in separately labeled Microsoft Word files in a computer. Subsequently, the coding framework and the relationships between the codes were analyzed and similar codes grouped into subthemes and main themes. The coding framework changed as the analysis process progressed, with similar themes and subthemes grouped together by the research team.

### 2.5. Trustworthiness

The researchers maintained data trustworthiness in the study by employing the concept of credibility, transferability, dependability, and confirmability [15, 16]. These concepts have been cited as the major criteria for establishing trustworthiness in qualitative research [15–18]. The researchers achieved transferability [19] in the study by giving a vivid description of the research setting and by employing a sample size large enough to yield data saturation. To achieve credibility of the data, the researchers purposefully recruited participants that met the inclusion criteria and who could provide in-depth information on their lived experiences with SCI. Again, the first author spent sufficient time in the field to gain a fuller and deeper understanding of participants' experiences. Credibility was further enhanced in the study through member-checking [18]; transcripts of the interview were taken back to the participants and explained to them in the local dialect for comments and verifications before conclusions were drawn from the data. In achieving dependability in the study, the researchers maintained an audit trail by giving a transparent and in-depth description of the research design, background of participants, and the methods used in collecting and analysing the data.

### 2.6. Ethical Considerations

The study was reviewed and approved by the Institutional Review Board of the Noguchi Memorial Institute for Medical Research, University of Ghana, with IRB number 00001276. It was subsequently approved by the research unit of the Tamale Teaching Hospital with reference number, TTH/R&D/SR/16/190. In addition, the purpose, objectives, and any potential benefits and risks for participating in the study were explained to participants in the local dialect (Dagbani) or English a few days prior to data collection. This allowed participants enough time to consider their participation. Participants who met the inclusion criteria and agreed to take part in the study were asked to give their consent by signing or putting their thumb print on the consent form. Participants were also informed that they could decline to participate or withdraw from the study even after they had signed the consent form without any consequences. Again, the permission to record the interviews was sought from each participant who agreed to participate in the study. All applicable institutional and governmental regulations concerning the ethical use of human volunteers were followed during the course of this research.

## 3. Results

### 3.1. Demographic Characteristics

The age range of participants was from 24 to 72 years with a mean of 50.5 years. Nine (69.2%) of the participants were males and four (30.8%) were females. Two of the participants were Christians (15.4%) and the rest were Muslims (84.6%); ten (77%) were married and three (23%) were single; eight (61.5%) had formal education and five (38.5%) had no formal education. Also, eight of the participants sustained their injury from road traffic accidents, three from tumour, and two from unestablished aetiologies. Participants have had their injuries for about 6 months to 5 years (Table 1).

In order to ensure anonymity and confidentiality, participants' real names have been replaced with pseudonyms in this manuscript. The three (3) main themes that emerged from the data were* “physical effects”; “psychological effects”; *and* “social issues” *related to their spinal cord injury.

### 3.2. Physical Effects: “I Am in Constant Pain”

The physical effects that participants endured following their injury were* pain, bladder and bowel problems, pressure ulcers,* and* neurological symptoms*.

All the participants in the study reported pain of varying degrees as part of the physical ramifications of their injury. Pain was the most devastating symptom they endured and it had a ripple effect on their mobility and functioning. Close to half of the participants reported that their pain had become chronic. Gariba described his pain in the following passage:

“My body was hot and very painful. The pain traveled along my spine down to my feet. Even until date, I still experience the pain in my body. When my wife just assists me to walk small, I feel severe pains around my neck…... It's not small.”

Mariam attributed her inability to perform her marital duties to the chronic pain she endures. She echoed:

“…..and then as a married woman too, because of the pain……your spouse, if not with understanding, he would not understand you. It would look as if you are neglecting him or something. He may not understand it is because of the pain, but you know the pain in which you are. Have you seen? The effect of the pain is not small.”

In addition to influencing their functioning, participants also reported that the pain they endured did not respond to pain medications. Alhassan described his experience:

“I'm still in pain till date, even as we speak. Every single blessed day I am in constant pain. Unless when am so engrossed in a conversation, that is the only time I don't feel the pain, otherwise I'm always in pain. I was given very strong pain killers but that couldn't stop the pain……so, I was advised to put a hold on them because of the fear of addiction.”

Also, as part of their physical experiences, some of the participants interviewed reported an inability to control their urine and feces following their injury. These participants identified the bladder and bowel incontinence as their most devastating health problem. It had a negative effect on their religious faith and social life. Adam had this to say:

“…..since the accident occurred, I have not been able to control my bowel and bladder. Both urine and feces come involuntarily. And that has been my biggest problem because…….you know cleanliness is next to godliness. As a Muslim, before you stand in front of your Maker, you should clean yourself yet, I can't hold it. It's so frustrating someone would always have to tidy you up.”

The participants with bladder and bowel incontinence also cited the high cost of dippers as some of the challenges they faced in the management of the bladder and bowel problems. Nana had this to say:

“…..and another difficulty is the cost of the diapers I buy, I have to be buying diapers all the time which is very expensive. The more I urinate, the more diapers I buy. So all that can be very very devastating.”

In addition to incontinence, a significant number of the participants reported experiencing pressure ulcers. The participants attributed the cause of their pressure ulcers to immobility and irregular turning in bed during hospitalization and, also, on the lack of health education prior to discharge related to care at home. Some of the participants had to live with the ulcers for months while others had to delay therapies to allow the pressure ulcers to heal. Peter shared his experience in the following passage:

“Because I could not be turned in bed regularly and whichever way I was positioned, I would be there until someone helps me turn to another position, my back and buttocks finally developed sores. The area around my buttocks got infected and formed a big sore and the nurses had to be dressing it every day until it got healed.”

Adam lived with pressure ulcers for almost 8 months. He blamed this on lack of health education by the health staff, prior to discharge. He had this to say:

“I developed some sores on my back and buttocks which lasted for close to 8 months because I was always lying on my back. And because I was not educated on the condition, and because my people too were not educated by the nurses on how to take care of me at home, I was always lying on my back and my relatives were not turning me in bed. I think that was why I developed the sores at my back.”

Another physical ramification reported by participants was neurological symptoms. The majority of the participants reported partial to flaccid paralysis of their upper and lower limbs and loss of sensation and stiffness following their injury. The participants also reported varying levels of immobility as a result of the paralysis and loss of sensations. Two (2) of the participants are still completely bedridden with no residual strength for mobility. Gariba is confined to a wheelchair for the rest of his life due to paralysis following his injury. He shared his experience;

“I could not feel any sensation in my lower limbs and I couldn't move my legs. Even till now I cannot walk. And when you prick me with a sharp object on my legs, I don't feel anything. I wonder if the sensation would ever come back and if I would be able to walk again.”

### 3.3. Psychological Effects “They See Me as a Disabled Person”

The psychological effects that participants endured as a result of the injury included* “anxiety”; “perceived physical disability”;* and* “self-adequacy.”*

The majority of the participants reported that they were saddened by the injury and the resultant consequences on their entire life. Some of these participants identified the sudden occurrence of the injury and the resultant paralysis and immobility as the major cause of their worry while others attributed their anxiety to the high cost of treatment and fear of remaining in a wheelchair for the rest of their lives. Peter had paralysis after his injury and was worried about the possibility of remaining in the wheelchair for life. He had this to share:

“I had seen it on TV where most people are confined to wheelchair as a result of this condition. So when I could not move my legs after the injury, I was wondering whether I was also going to be in that same situation. In fact that one alone gave me some psychological torture and I was really worried.”

Alhassan also lamented the suddenness of his condition and its negative impact on his life and job. He shared his experience:

“I was deeply saddened by…….the way the whole thing happened. I mean, elm……. you are healthy and all of a sudden, you can't even move your legs, you can't do anything. Even till date, I can no longer live the life I used to live, and my job is something else all because of this injury. That is really worrying.”

In addition to the anxiety, participants with paralysis and complete immobility also reported that society now perceived them as disabled persons as a result of their physical limitations. The participants further reported that they received favors from people especially during functions because of the societal perception of them.

Zack shared his ordeal:

“I would say society now perceives me as a physically challenged person because I can't walk. Even till date, they still see me as weak and feeble, to the extent when I'm seated with friends in the cold weather, you would hear some of them urging me to better go inside because they think I'm sick and the weather is not good for me.”

Peter also believed that society sympathized with him wherever he went because they saw him as a disabled person. He noted:

“Let me say, the society sympathizes with me anywhere I find myself. For instance, the last time we went for a program, and then an elderly man was seated. Once he saw me coming, he just got up and gave me his seat, and I realized it was because of the crutches I was using. And usually that doesn't happen. It is supposed to be the young one giving the seat to the elderly person. So, I would say they have been sympathetic because they see me as a disabled person.”

Participants reported various levels of self-adequacy with some expressing higher morale and perceived abilities to return to work than others. It was revealing to note that employment status rather than the physical health of the participants appeared to contribute to higher self-adequacy beliefs of participants. Participants with gainful employment or on sick leave with pay expressed more desire and willingness to resume work than those without employment. Adams was a teacher before his injury rendered him bedridden. He wanted to go back to the class room because he still believed he had something to offer society despite his complete paralysis and immobility. He said:

“I have realized that there is still something in me that I am denying the society of. So I want to go out there and see whether I can make a change, because you don't know what you can do until you start doing it. So I want to go back to the class room and see what I can do again.”

In contrast to the above, participants without jobs after the injury expressed less motivation to return to work or secure a new job, regardless of their physical state of health. Although Zack had regained his mobility at the time of the interview, he still felt he was not fit enough to take up a job and depended on his sons for living. He lamented;

“My life has become like that of a dependent and I wonder if I could ever be productive in life again. I have never been able to take any job since the injury because am still not strong. So, I'm always here, I don't go anywhere and I depend largely on my sons for living.”

### 3.4. Social Issues “Everyone Has Abandoned Me”

The participants reported a number of societal factors including support, neglect, marital and spousal problems, and impaired participation that promoted or hindered their adjustment to the injury.

The majority of the participants received some form of support from family, friends, spouses, and institutions. They acknowledged that the support received from these sources contributed significantly to their recovery and adjustment to the injury. Among the married participants, spousal support was identified as the most important form of support and took primacy over other forms of support. The majority of the unmarried participants identified support from friends and family as the most valuable form of support they received. A few of the participants also reported that the ties between them and their families deepened in the aftermath of their injury. Sule identified his wife as his main source of support and inspiration. He shared the following:

“It is only my second wife who still hopes that, God can still perform His miracle and I would be able to walk again. She is my only hope and the only form of support left for me. Without her I wouldn't know what to do because she cooks for me, baths me, and does everything for me.”

Adam was a single 31-year-old man. He believed he had been lucky with friends:

“I must say that I have been lucky with friends. With this condition, friends have been of immense support to me. My relationship with my friends has increased after the condition. Even those I never considered my close friends have now become closer after the injury. So, in general, I would say my friends have been good to me to the extent some of them are now like brothers to me.”

A few of the participants reported that they had been jilted and neglected by family members and loved ones following their injury. These participants questioned why family relatives and loved ones could turn their back on them simply because they suffered a SCI. Some of the participants mentioned that just a few of their friends and relatives still remembered them occasionally, while a few others had completely lost touch with family and friends after the injury.

Gariba had been neglected by both family and friends. He expressed his sorrow in the following passage:

“Before the injury I was well to do and had many friends because many were those who followed me because of their stomach. But right after the injury, they all ran away. There has never been a time that even a single one of them would call. I really don't know whether because they think I would demand something from them or not but none of them still visits me. They wouldn't even call.”

He continued:

“My family too, everyone has abandoned me. I come from a very large extended family. But since I came to this village for herbal treatment, not even a single member of my family has been here on a visit. They have all given up on me because they believe I can never get back on my feet again.”

Quite a number of the participants reported suffering romantic disappointment after their injury, with the majority of the unmarried participants reporting more romantic disappointments from their fiancés than the participants that were married. Alhassan was jilted by his fiancé in a relationship that took him close to eight years to build. He narrated:

“I had a childhood girlfriend whom I began dating way back in senior high school. But exactly a month after the injury this girl left me, after going out with me for close to 8 years. And her going actually shocked me because I had never dated a woman in my life and after 8 years, we never quarreled, I only got involved in an accident, and just because of that, this lady called it quits.”

Some of the participants who were single expressed interest in marriage but cited societal views and financial constraints as some of the reasons that keep them from venturing into marriage. Adam lamented:

“There are so many things that prevent me from venturing into marriage. One of them is societal views or expressions. The moment you venture into it, society would say you are still sick and instead of thinking of how to get well, you are rather thinking of women, forgetting that, may be if I had a woman by my side, she would have reduced some pressure on me. The other reason is about finances, because if I want to marry, I cannot be in this room. I would need a place where I can feel very comfortable and convenient, which I cannot afford. Those are my reasons for being afraid to enter into marriage.”

In addition to romantic disappointments, four of the participants (3 males and 1 female) reported an erectile or sexual dysfunction after the injury. Two of the participants attributed their sexual dysfunction to the chronic pain they endured after the injury. Wumbei shared his experience:

“It's just the pain. Even in bed, I no longer perform as I used to. It's just occasionally that I would be able to perform and that is my major problem because my wives wouldn't understand even if I told them the truth. I know they are not happy about that, but I know very well it's all as a result of the injury.”

Mariam also attributed her inability to perform her matrimonial duties to the chronic pain she endured. She explained:

“And then as a married woman too, your spouse……. if not with understanding, he would not understand you. It would look as if you are neglecting him or something, but you know the pain in which you are. Have you seen?”

In addition, the participants in the study also verbalized that they were unable to partake in physical activities and other social functions such as weddings, funerals, and naming ceremonies. They cited challenges with mobility, pain, and bladder and bowel incontinence as the reasons for their inability to exercise and to attend social functions.

Mariam had to stop attending social gatherings because of her difficulties with mobility. She narrated:

“On social issues, you know we ladies, every weekend, outdooring, wedding and what not……. now I don't participate in any because, I can't walk because of the pain. So it has affected me a lot.”

Adam had bowel and bladder incontinence and as a result he did not mingle with people because of fear of embarrassing himself in public. He bemoaned:

“You know as a person, personal hygiene is very important. So because of my inability to control my urine and feces, I cannot associate with people even if I wish to. Because, you know both urine and feces come with smell and you can be with people and all of a sudden you soil yourself.”

## 4. Discussion

To the best of our knowledge, this was the first study that explored the lived experiences of persons with SCI in Ghana. The findings revealed that persons with SCI suffer physical manifestations such as chronic pain, bladder and bowel incontinence, immobility, pressure ulcers, and neurological symptoms that have enormous consequences on their functioning and general wellbeing. These findings concur with other studies that examined the experiences of persons with SCI [3, 20–22]. Pain was one physical symptom that was reported by all the participants of the present study. The participants verbalized varying degrees of pain, with the majority of them reporting that their pain had lasted for almost five years. Previous studies have also identified pain as the most prevalent symptom among persons with SCI [23–27]. While our finding concurs with the findings of most previous studies, the occurrence and prevalence of pain in SCI seem to vary slightly across geographical locations. For instance, while most previous reports estimate a pain prevalence of more than 60% among persons with SCI [23–25, 28], Rubinelli and colleagues [29] assert that the prevalence of pain among Swiss nationals with SCI is 29.70% [29]. Future systematic reviews involving data from across the globe would help throw more light on differences in the prevalence of pain in SCI across various geographical locations.

The majority of participants of our study identified pain as the most devastating health problem that negatively affected their mobility and functioning. This finding again adds to the ongoing debate on the effects of pain on the functioning among persons with SCI. While in tandem with our findings, several studies have reported a significant relationship between pain intensity and activity of daily living among persons with SIC [20, 30, 31]; Ullrich et al. [26] also assert that there is no significant relationship between pain intensity and overall pain interference in persons with SCI. Differences in demographic characteristics of participants and the use of varied research designs by scholars may account for the above differences in findings. Future studies involving large transcultural samples may help shed light on the effects of pain on the functioning of persons with SCI.

In addition to the negative effects of pain on the functioning of participants of the present study, a number of the participants also reported that their pain was not responsive to pain medications that were prescribed for them at the hospital. This compelled some of the participants to abandon their pain medications and to seek other remedies in dealing with their pain. In support of this finding, Henwood and colleagues [32] also reported that pain medications are often inadequate in relieving neuropathic pain in SCI and the presence of unbearable side effects from these medications often lead to the decision to stop the medication and proceed to the next available option. Clinicians should therefore focus on other approaches to pain management in SCI such as acupuncture and coping strategies which have demonstrated to be effective in the management of pain in SCI [33–35]

More so, our participants reported bladder and bowel incontinence as part of their physical experiences, with a significant negative effect on their participation. A few of the participants verbalized that they were unable to socialize with others in society and were unable to meet their religious obligations as a result of the bladder and bowel incontinence. Previous similar studies have also reported on the negative effect of bladder and bowel problems on the social life of persons with SCI [20, 36, 37]. Again, the majority of participants with bladder and bowel problems in our study used diapers in the management of their bladder and bowel incontinence. This finding differs significantly from previous reports which identified digital rectal stimulation, the use of enemas, abdominal massage, Valsalva maneuver, and the use of indwelling catheters as the most widely used and effective approaches to the management of bladder and bowel incontinence among persons with SCI [22, 38, 39]. Inadequate predischarge health education on the continuity of care at home and the lack of bladder and bowel management programs for spinal injured persons at the research setting [40] may account for the differences in findings between our study and that of previous studies [38, 39].

To add to the above, a few of the participants in the present study also developed pressure ulcers after their injury. The participants reported that they developed the pressure ulcers while on admission at the hospital. Contrary to the current finding, Gould and colleagues [41] reported that majority of persons with SCI developed pressure ulcers as outpatients, while residing in the community. Irregular turning of patients in bed and poor quality health care at hospitals at the research setting due to inadequate staffing might have accounted for the difference in findings between the present study and that of Gould et al.'s [41] study. Neurological symptoms and their impact on functioning were also reported in the present study, as part of participants' physical experiences with SCI. The neurological symptoms that were reported by the majority of the participants included partial to flaccid paralysis of lower and/or upper limbs, stiffness of lower and/or upper limbs, and loss of sensations. Several systematic literature reviews have also identified paralysis, stiffness, and loss of sensation as some of the neurological manifestations of SCI [42–44]. The participants attributed their immobility and self-care deficits to the neurological problems and their neurological deficits and this agrees with the findings of Birns and Fitzpatrick [45]. According to Birns and Fitzpatrick [45], spasticity can cause pain, stiffness, and spasms that have a negative impact on the overall functioning of the individual.

Besides the physical ramifications of SCI, the findings of the present study also revealed a number of negative psychological outcomes as reported by the participants. These psychological outcomes included anxiety, perceived disability, and low self-efficacy. In narrating their anxiety, some of the participants identified the sudden onset of their injury and the accompanying symptoms as the major cause of their anxiety. A few participants attributed their anxiety to fear of remaining in a wheelchair for the rest of their lives, while others identified the loss of their businesses after injury and the resultant financial difficulties as the main source of their anxiety. High levels of anxiety among persons with SCI have been reported in previous studies [46, 47]. In a systematic review of psychological morbidity in SCI, Craig et al. [48] observed that people with SCI have higher risks for anxiety disorders, elevated levels of anxiety, and poor quality of life. In addition, participants without gainful employment or reliable sources of income in the current study reported low levels of self-efficacy towards work or employment than those with gainful employment. The participants (without employment) expressed less desire or unwillingness to return to work or get a new job, regardless of their physical abilities or level of recovery. On the other hand, the participants with gainful employment or paid sick leave, expressed a desire to resume work or start a trade regardless of their physical state of health and did not recognize their disability as a disincentive to work. This finding differs from previous studies that identified age and psychological variables as the determinants of employment self-adequacy among persons with SCI [49, 50]. Future studies with large samples and using different methodologies would shed more light on the determinants of employment self-adequacy and self-efficacy beliefs among persons with SCI.

The participants also shared a number of societal related factors that promoted or hindered their adjustment to the injury. These factors included “support,” “neglect,” “marital and spousal problems,” and impaired participation. As a result of the debilitating physical symptoms that participants endured, the majority of them sought the support of family members, friends, and spouses, to adjust to their injury and to meet their basic needs. The participants acknowledged that the support they received impacted positively on their adjustment to the injury. The need for social support for persons with SCI is well documented in the literature [51, 52]. Spousal support was identified by the participants who were married as the most important form of support, while the majority of the unmarried participants identified support from friends and family support as the most valuable form of support. Similarly, Kalpakjian et al. [53] reported on the importance of marriage to the wellbeing of persons with SCI and argued that being married was a facilitator for lower depressive symptoms and wellbeing among persons with SCI [53]. Similarly, in a cross-sectional survey, Tramonti and colleagues [52] identified couple and family support as the most important sources of support when compared with support from informal social networks such as friendships [52]. This implies that spousal and family support play a pivotal role among persons with SCI in the adjustment and adaptation to the injury.

Although the majority of the participants received support, some other participants reported being neglected and jilted by their friends, families, and loved ones following their injury. Some of the participants revealed that the bond that existed between them and their family members and friends had deteriorated following the injury. Similarly, in a study to assess the impact of SCI on South African youth, Njoki et al. [54] identified diminished intrapersonal and interpersonal relationships as some of the ramifications of SCI. Besides the familial neglect, a number of the participants also reported being jilted by their wives or fiancés following their injury. The majority of participants that reported spousal disappointment belonged to the unmarried class of participants who were courting before the injury. In line with this finding, divorce prevalence is 1.80 to 2.07 higher among persons with SCI than in the general population according to literature [55]. Again, some of the participants of our study reported an impairment in sexual activity as a result of erectile dysfunction. Several other studies have reported on the high prevalence of erectile dysfunction and sexual dissatisfaction among persons with SCI [56–58]. Erectile dysfunction or impairment in sexual activity was identified in our study, as a risk factor for spousal disappointment. Nearly all the participants that reported erectile dysfunction/sexual impairment also reported to have suffered some form of spousal disappointment or divorce. This finding is not in consonance with previous reports on the risk factors for spousal separation after a SCI [59]. In a systematic literature review Kreuter [59] identified being young, being female, being black, being nonambulatory, and having no children as the risk factors for divorce and romantic disappointment among persons with SCI [59]. Differences in sociodemographic characteristics between our sample and that of previous studies might have accounted for the differences in findings.

On social participation, the majority of the participants of the current study verbalized that they were unable to partake in important social events such as weddings, outdoorings, funerals, and other festive occasions that are common in Ghanaian society. Some of the participants cited bladder and bowel incontinence as the major physical health problem that hindered their socialization and participation in social events while others mentioned immobility and chronic pain as the factors that limited their participation in social functions. This finding is consistent with previous studies on participation in SCI [20, 60, 61]. Bloemen-Vrencken et al. [20] revealed that the impact of bladder and bowel problems on social activities is considerably higher than their impact on daily activities in persons with SCI. Also, Williams et al. [62] completed a metasynthesis and reported loss of bodily control, fatigue, and secondary conditions as some of the factors that prevent leisure time physical activity among persons with SCI.

## 5. Conclusion

The findings suggest that spinal cord injury is a life threatening condition and that persons with the condition grapple with a myriad of physical symptoms ranging from chronic pain and paralysis of lower and/or upper limbs, to bladder and bowel incontinence. These physical symptoms impact negatively on the psychosocial life of affected persons and their overall quality of life. Future research involving larger samples and employing different methodologies would further illumine our understanding of the experiences of persons with SCI and the impact of such experiences on their overall functioning and quality of life.

## Figures and Tables

**Table 1:** 

**Demographic characteristic**	**Average**	**N (**%**)**
**Age**	50.5	
< 40 years old		4 (30.8%)
≥ 40 years old		9 (69.2%)
**Sex**		
Male		9 (69.2)
Female		4 (30.8%)
**Religion**		
Muslim		11 (84.6%)
Christian		2 (15.4%)
**Marital status**		
Married		10 (77%)
Single		3 (23%)
**Level of Education**		
Educated		8 (61.5%)
No Formal Education		5 (38.5%)
**Duration of Injury**		
< 2 years		3 (23%)
≥ 2 years		10 (77%)

## Data Availability

The data used to support the findings of this study are available from the corresponding author upon request.
